# Supplementary material for: Identification and validation of a novel signature for prediction the prognosis and immunotherapy benefit in bladder cancer
Source: PeerJ. 2022 Jan 25;10:e12843. doi: 10.7717/peerj.12843 (PMC8796709; doi:10.7717/peerj.12843)
Supplement: Supplemental Information 1 [file peerj-10-12843-s001.docx]

**Supplementary Table S1**. General information for Gene Expression Omnibus (GEO) bladder cancer datasets

| GEO ID | Number of | Number of | Subtype | Gender | Survival outcome | Platform | Country |
| --- | --- | --- | --- | --- | --- | --- | --- |
|  | Noncancerous | Cancer |  |  |  |  |  |
|  | Bladder Tissue | Tissue |  |  |  |  |  |
|  | Samples | Samples |  |  |  |  |  |
| GSE121711 | 10 | 8 | NMIBC:5; MIBC:3 | Female:2; Male:14 | PFS; RFS | GPL17586 | Spain |
| GSE40355 | 8 | 16 | NMIBC:13; MIBC:3 | Female:2; Male:14 | OS | GPL13497 | Germany |
| GSE3167 | 14 | 46 | - | - | - | GPL96 | Denmark |
| GSE37815 | 6 | 18 | NMIBC:16; MIBC:2 | Female:2; Male:16 | OS; PFS | GPL6102 | South Korea |
| GSE13507 | 68 | 165 | NMIBC:103; MIBC:62 | Female:30; Male:135 | OS; PFS | GPL6102 | South Korea |

NMIBC: Non-muscular invasive bladder cancer, PFS: Progression-free survival, OS: Overall survival.
