# Supplementary material for: Identification and validation of a novel signature for prediction the prognosis and immunotherapy benefit in bladder cancer
Source: PeerJ. 2022 Jan 25;10:e12843. doi: 10.7717/peerj.12843 (PMC8796709; doi:10.7717/peerj.12843)
Supplement: Supplemental Information 2 [file peerj-10-12843-s002.docx]

**Supplementary Table S2**. Sequences of primers used in the present research.

| **Gene name** | **Primer sequence (5' to 3')** | **Product length (bp)** |
| --- | --- | --- |
| CNKSR1-F | AAGCCTGACAGAGGGACTTCT | 96 |
| CNKSR1-R | AGGACATCAATAGGGGTCTTGG |  |
| COPZ2-F | ATTGTGGATGGCGGTGTGAT | 102 |
| COPZ2-R | GGCCACACTCTGTTCAGTCA |  |
| CXorf57-R | TCCTGTGCATAGATAACGTCCA | 130 |
| CXorf57-F | GCCAGGTAATGACTCTTCCCG |  |
| FASN-F | CCTGGCTGCCTACTACATCG | 102 |
| FASN-R | CACATTTCAAAGGCCACGCA |  |
| PCOLCE2-F | TGGCACATTGTAGCCCCAAA | 110 |
| PCOLCE2-R | CCGCCATTAAACACAGCCAC |  |
| RGS1-F | CCTGGTGGGCAATGAACAG | 138 |
| RGS1-R | TTGGCTGAGATTCGTACCACG |  |
| SPINT1-F | AACTACCTCACGAGGGAAGTG | 110 |
| SPINT1-R | GGTTGTACCTTCAAGTCTATGCC |  |
| TPST1-F | TTTCTAGGTTATTCCCCAATGCC | 146 |
| TPST1-R | AGCACGATTCCACTTTGTCAA |  |
| GAPDH-F | AGAAGGCTGGGGCTCATTTG | 136 |
| GAPDH-R | GCAGGAGGCATTGCTGATGAT |  |
