# Supplementary material for: Identification and validation of a novel signature for prediction the prognosis and immunotherapy benefit in bladder cancer
Source: PeerJ. 2022 Jan 25;10:e12843. doi: 10.7717/peerj.12843 (PMC8796709; doi:10.7717/peerj.12843)
Supplement: Supplemental Information 4 [file peerj-10-12843-s004.docx]

**Supplementary Table S4**. Detailed information of the current immune-related signature model for BC.

| Cohorts | AUC | | Immune-related signature | | | | | | |
| --- | --- | --- | --- | --- | --- | --- | --- | --- | --- |
|  | |  | Our signature | PMID:32841548 | PMID:32117435 | PMID:32096345 | PMID:32763782 | PMID:33048468 | PMID:32607061 |
| Training Cohort | 1-year OS | | 0.771 | 0.697 | 0.711 | ≈0.700 | - | 0.74 | 0.666 |
|  | 3-year OS | | 0.735 | 0.721 | 0.754 | ≈0.700 | 0.715 | 0.75 | 0.657 |
|  | 5-year OS | | 0.718 | 0.743 | 0.772 | ≈0.700 | 0.704 | 0.76 | 0.652 |
| Validation Cohort | 1-year OS | | 0.677 | 0.651 | 0.646 | - | - | - | 0.667 |
|  | 3-year OS | | 0.694 | 0.665 | 0.645 | - | - | - | 0.638 |
|  | 5-year OS | | 0.676 | 0.634 | 0.625 | - | - | - | 0.647 |
| Immunotherapy prediction |  | | Yes | Yes | - | Yes | - | - | Yes |
|  |  | | 0.647 | 0.655 | - | 0.64 | - | - | 0.584 |
