# Supplementary figures and images for: Identification and validation of a novel signature for prediction the prognosis and immunotherapy benefit in bladder cancer
Source: PeerJ. 2022 Jan 25;10:e12843. doi: 10.7717/peerj.12843 (PMC8796709; doi:10.7717/peerj.12843)

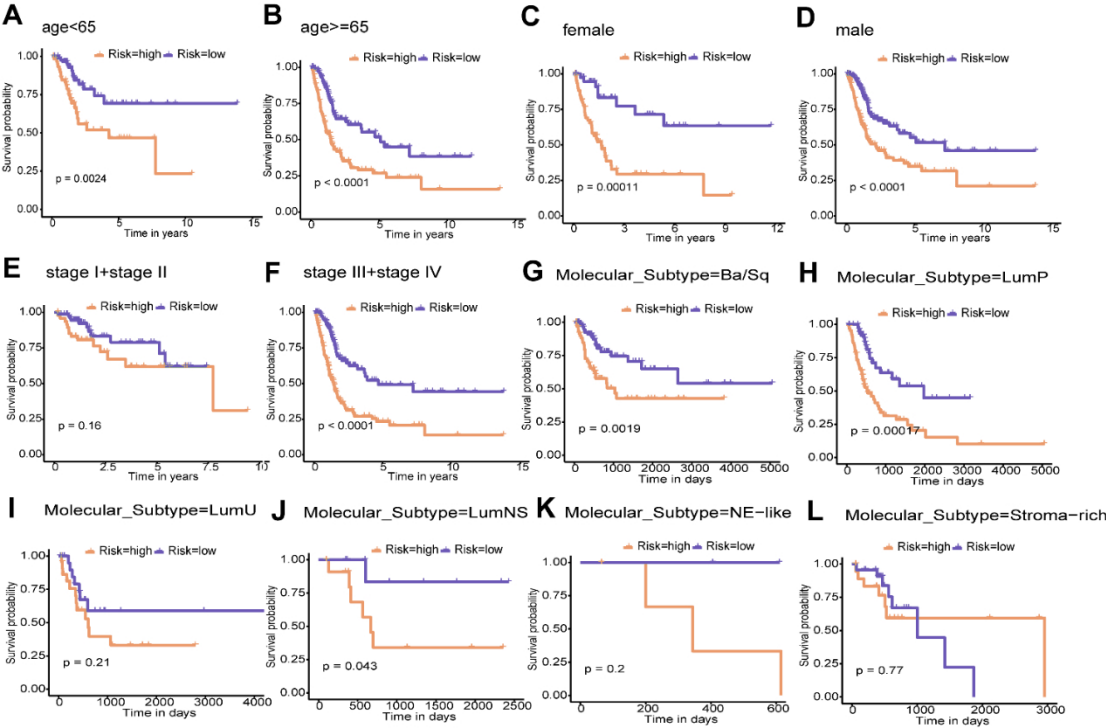

Supplement: Supplemental Information 5 — (A) Age <65 years, (B) age ≥65 years, (C) female, (D) male, (E) AJCC stage I–II, (F) AJCC stage III–IV, (G) molecular subtype of basal/squamous, (H) molecular subtype of luminal papillary, (I) luminal unstable (J) luminal non-specified (K) neuroendocrine-like (L) stroma-rich [file peerj-10-12843-s005.pdf]

**A****CNKSR1**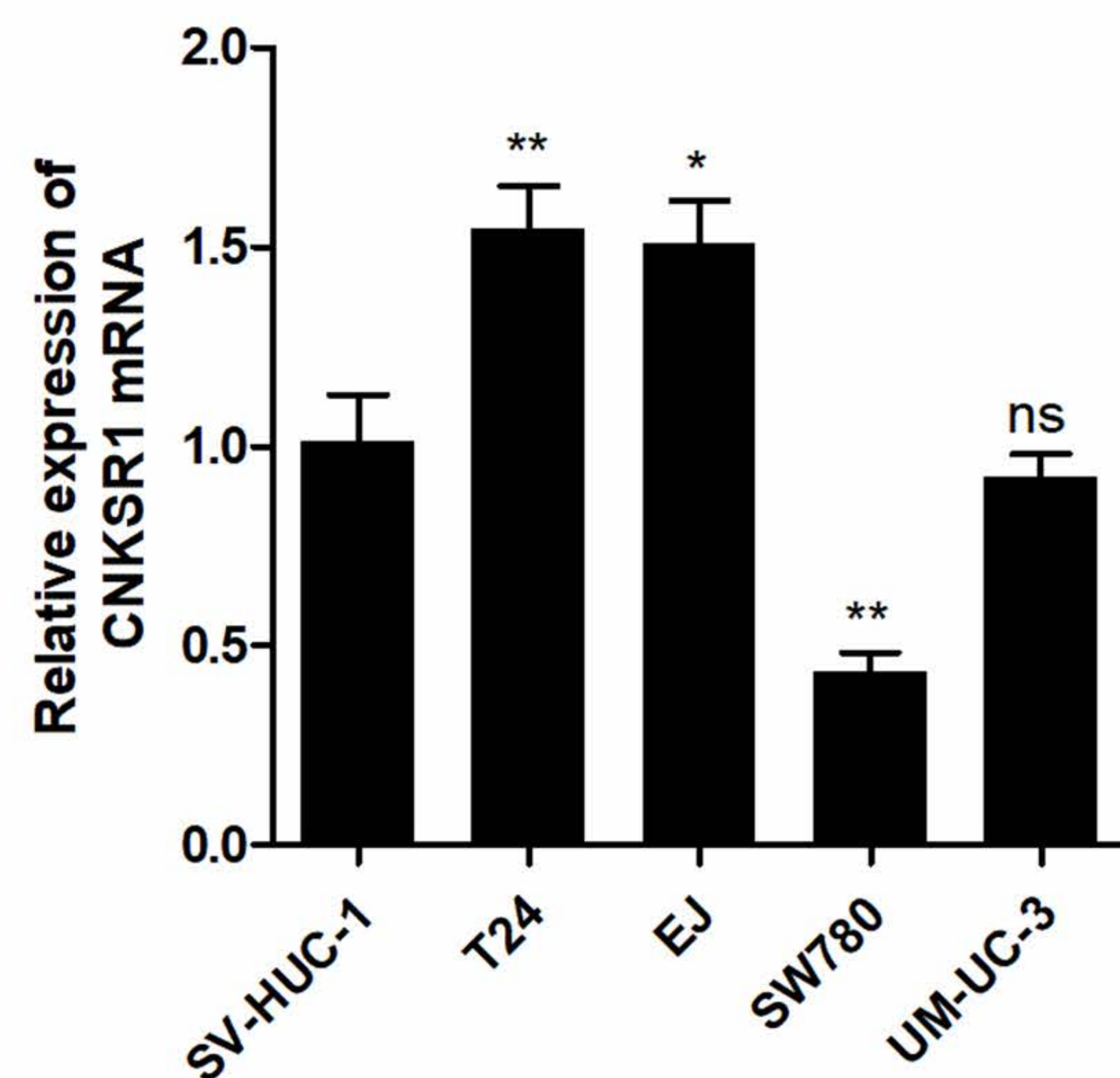**B****COPZ2**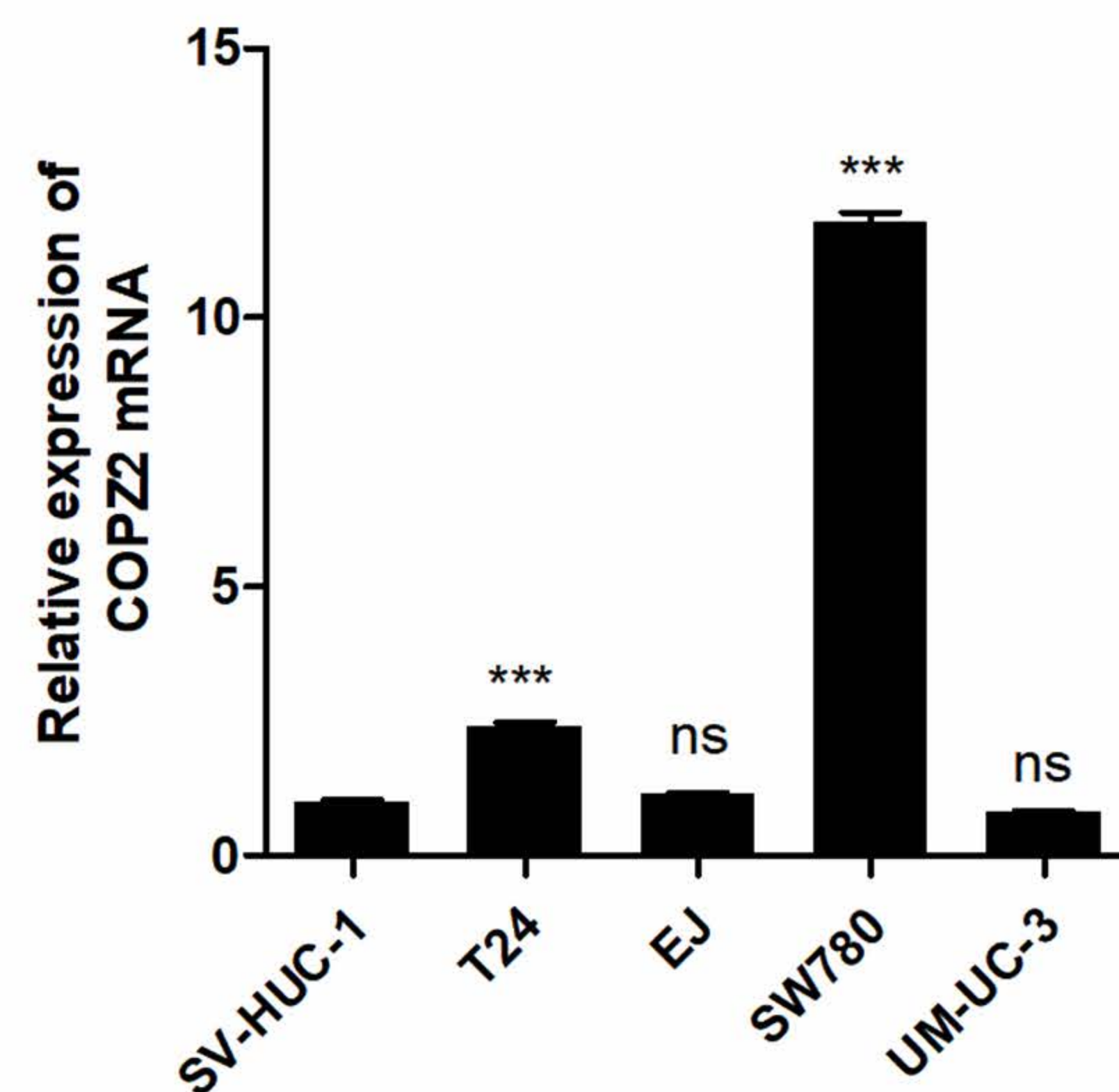**C****CXorf57**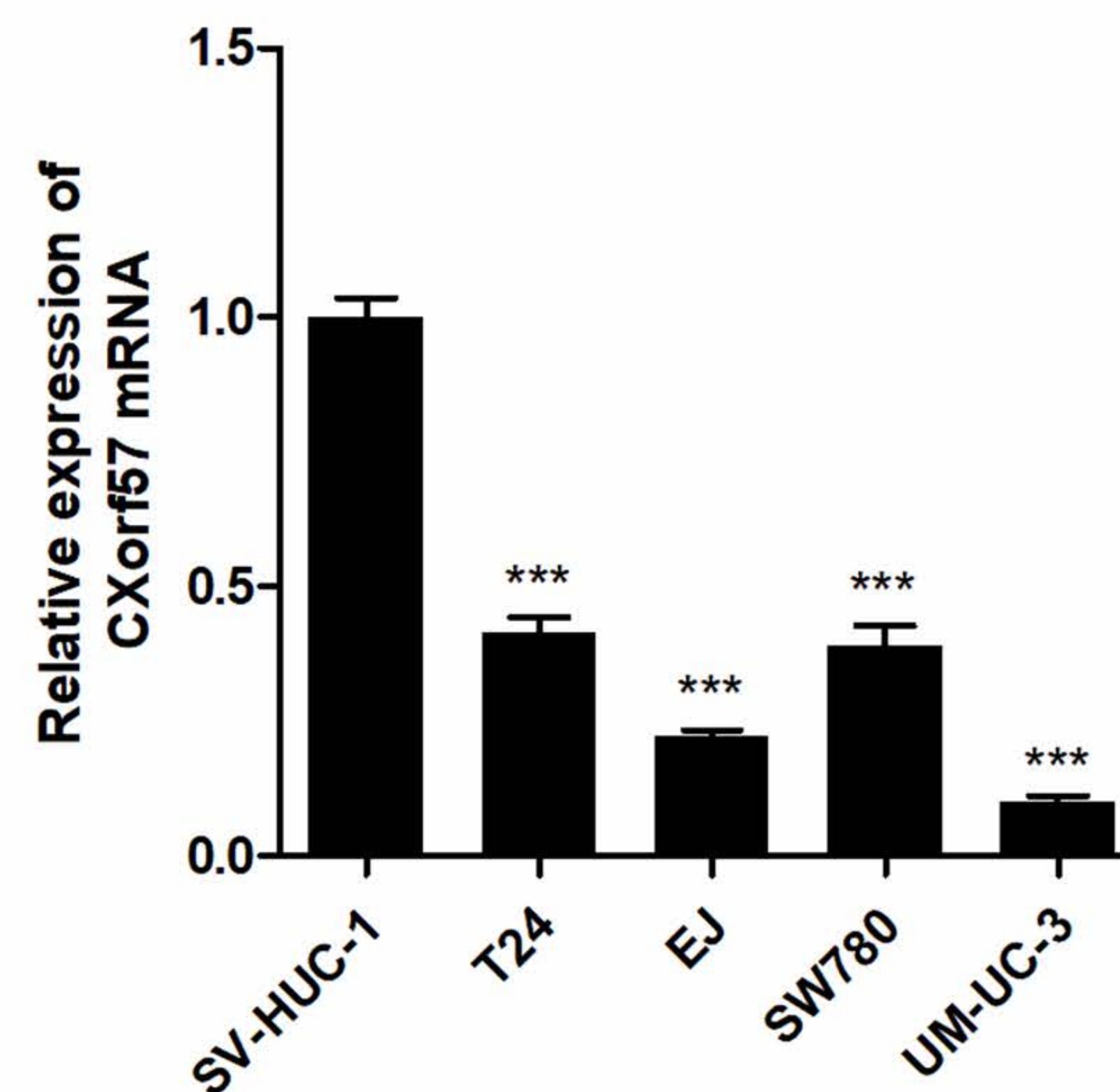**D****FASN**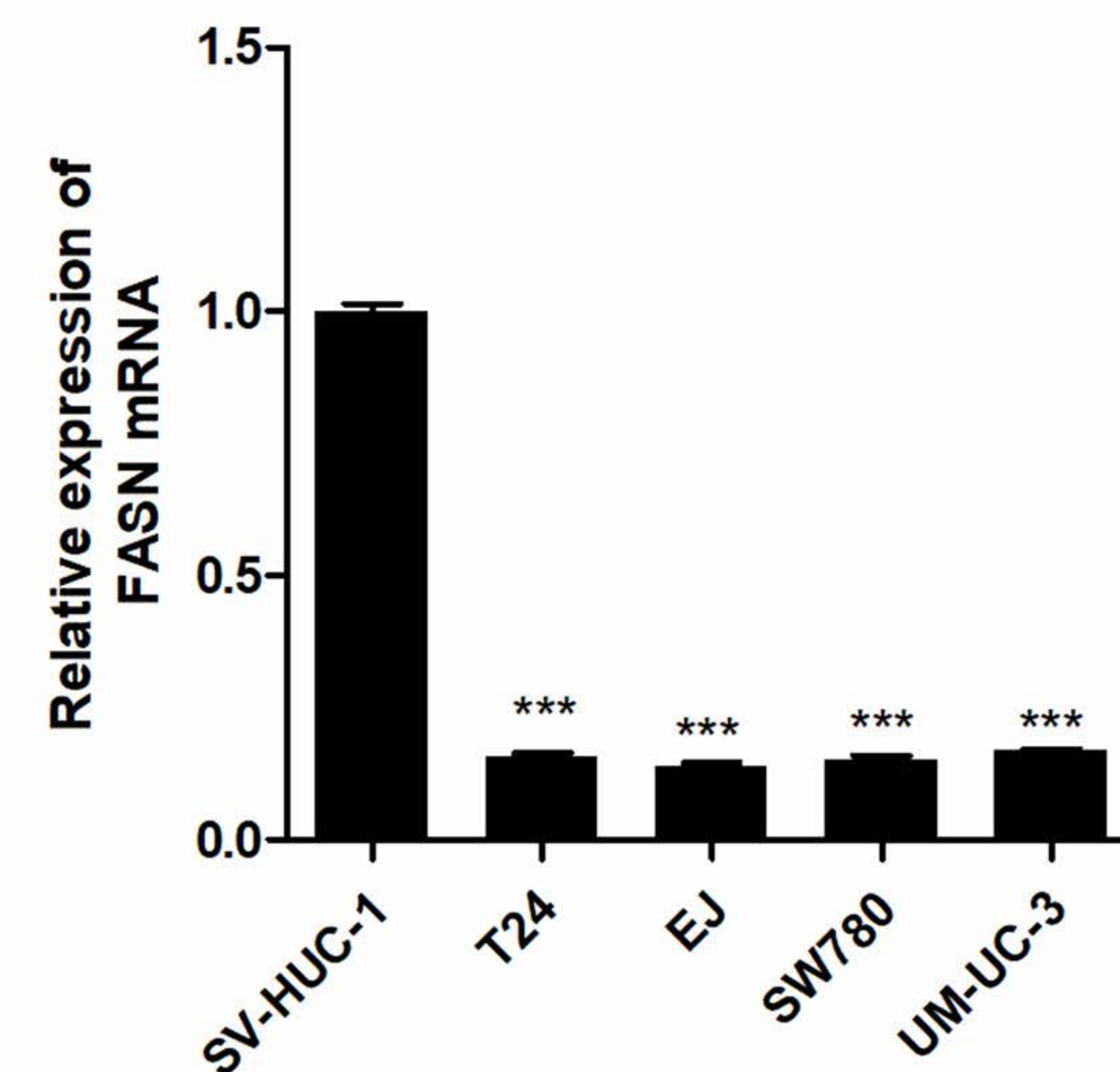**E****PCOLCE2**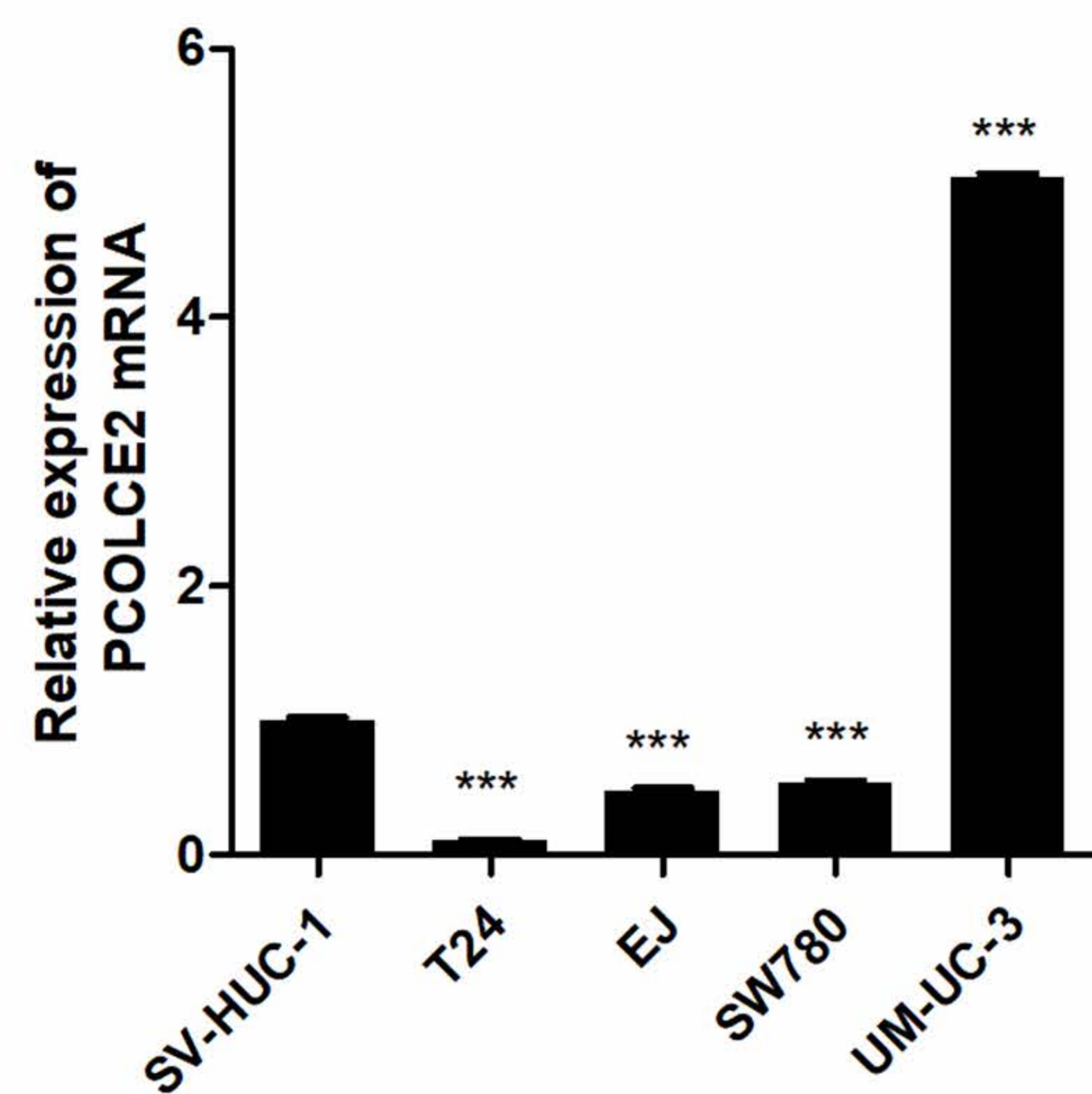**F****RGS1**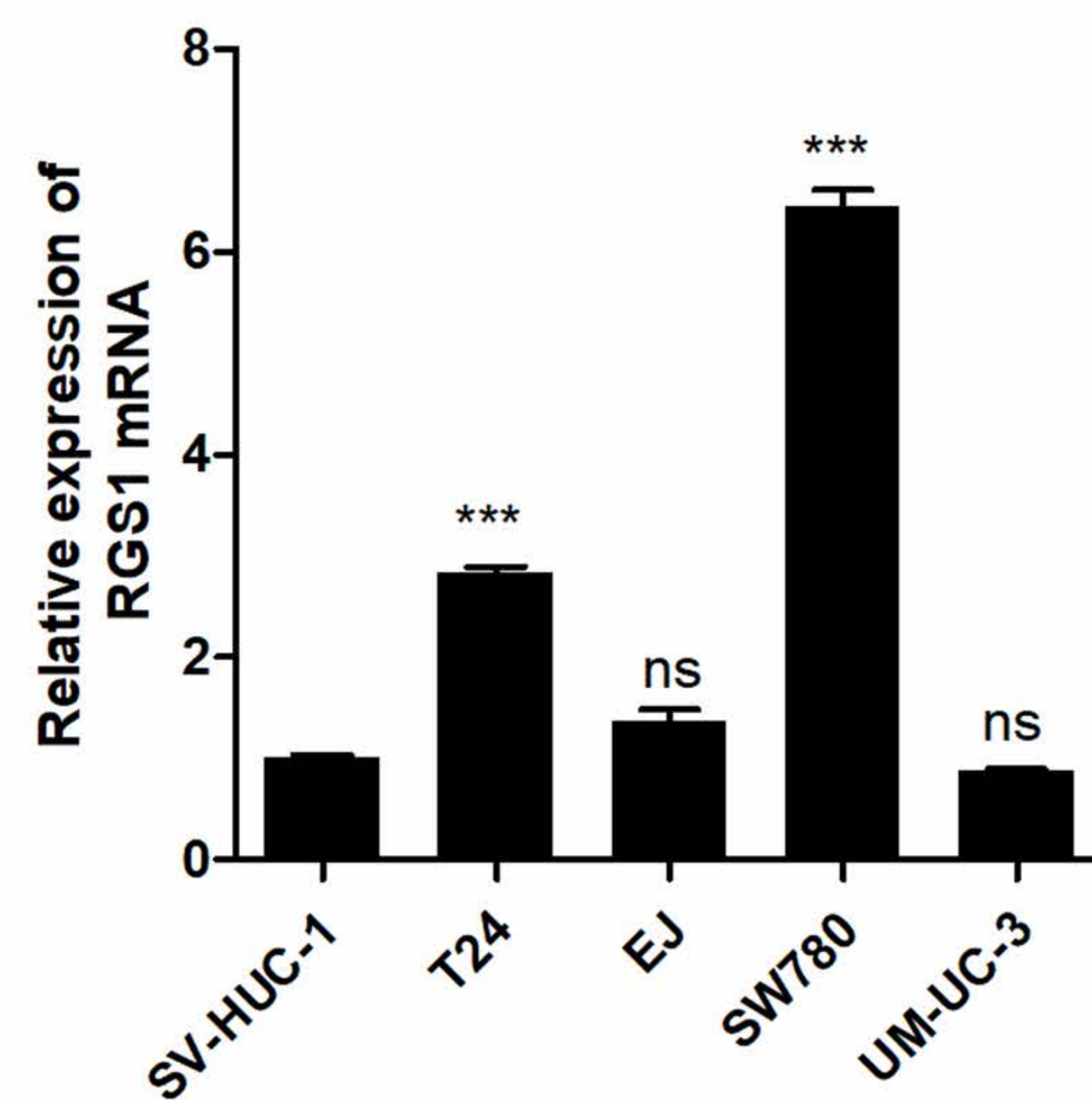**G****SPINT1**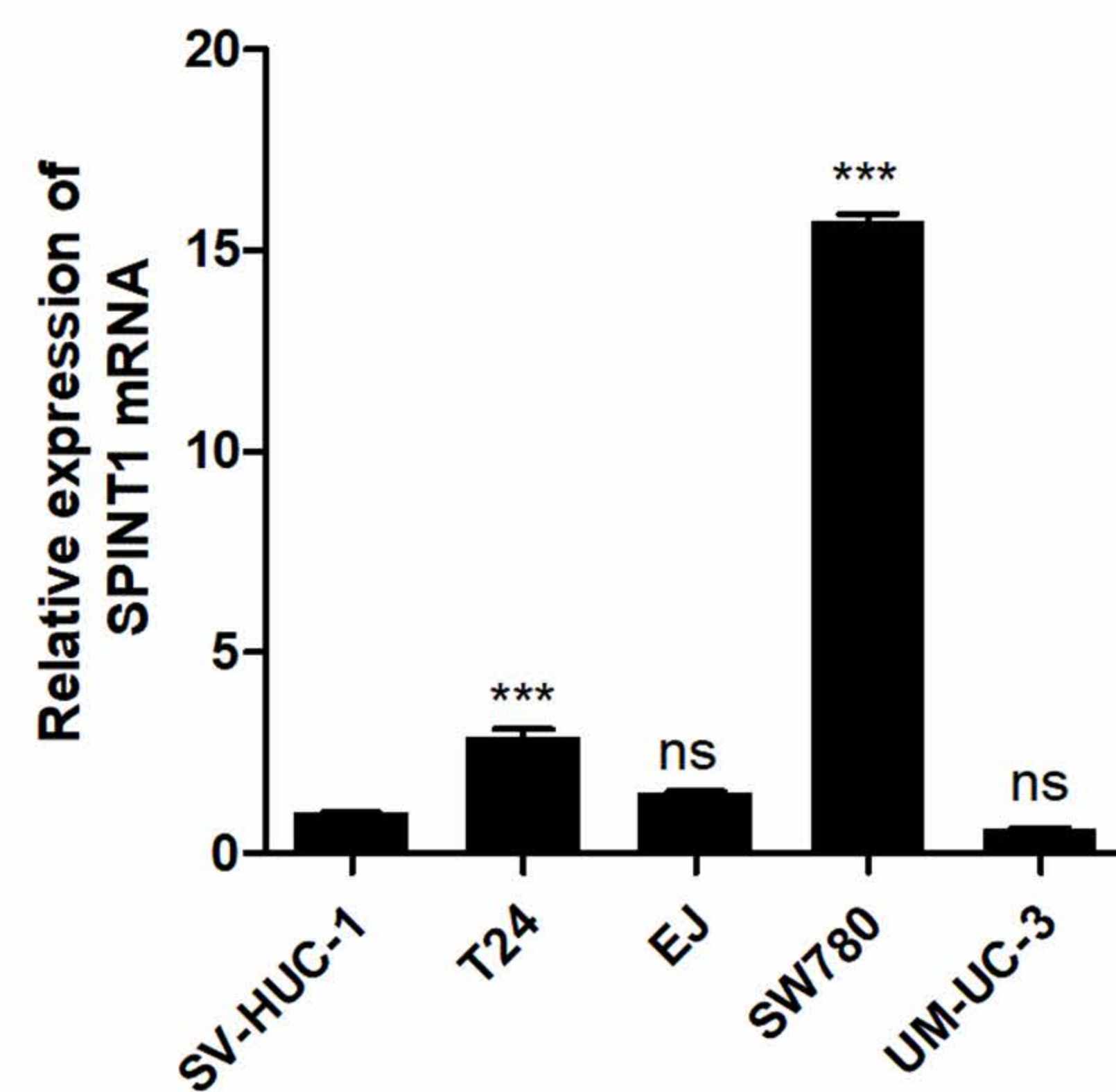**H****TPST1**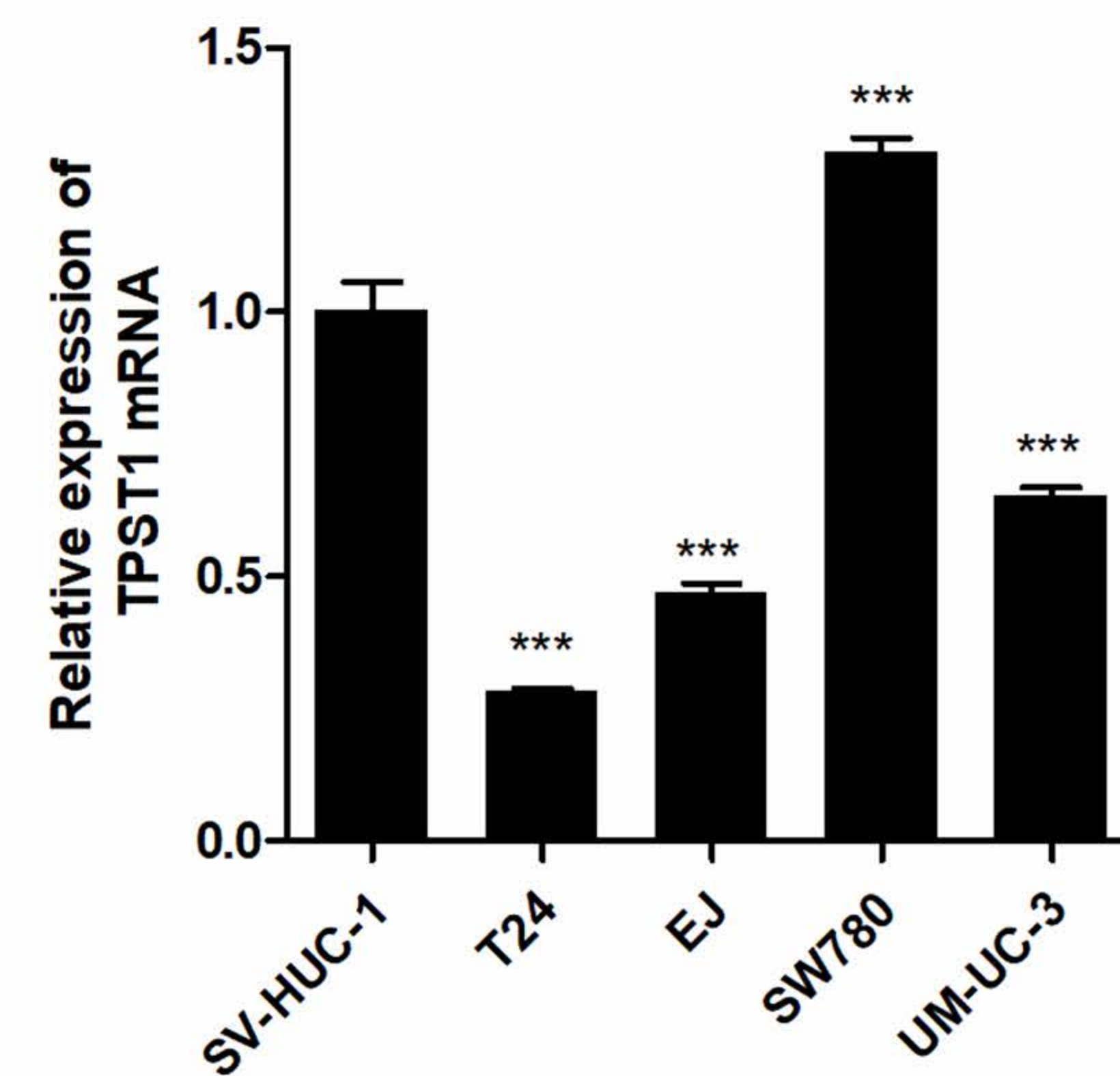

Supplement: Supplemental Information 8 — qRT-PCR for expression of qRT-PCR detection of the 8 crucial genes expression in BC cells (EJ-1, T24, UM-UC-3, SW780) and SV-HUC-1 cells (the normal human urinary tract epithelial cells). *P < 0.05, **P < 0.01, ***P < 0.001. (A) CNKSR, (B) COPZ2, (C) CXorf57, (D) FASN, (E) PCOLCE2, (F) RGS1, (G) SPINT1, (H) TPST1. ns, not significant. [file peerj-10-12843-s008.pdf]
