# Supplementary material for: Identification and validation of a novel signature for prediction the prognosis and immunotherapy benefit in bladder cancer
Source: PeerJ. 2022 Jan 25;10:e12843. doi: 10.7717/peerj.12843 (PMC8796709; doi:10.7717/peerj.12843)
Supplement: Supplemental Information 6 — Images were taken from the Human Protein Atlas [file peerj-10-12843-s006.pdf]

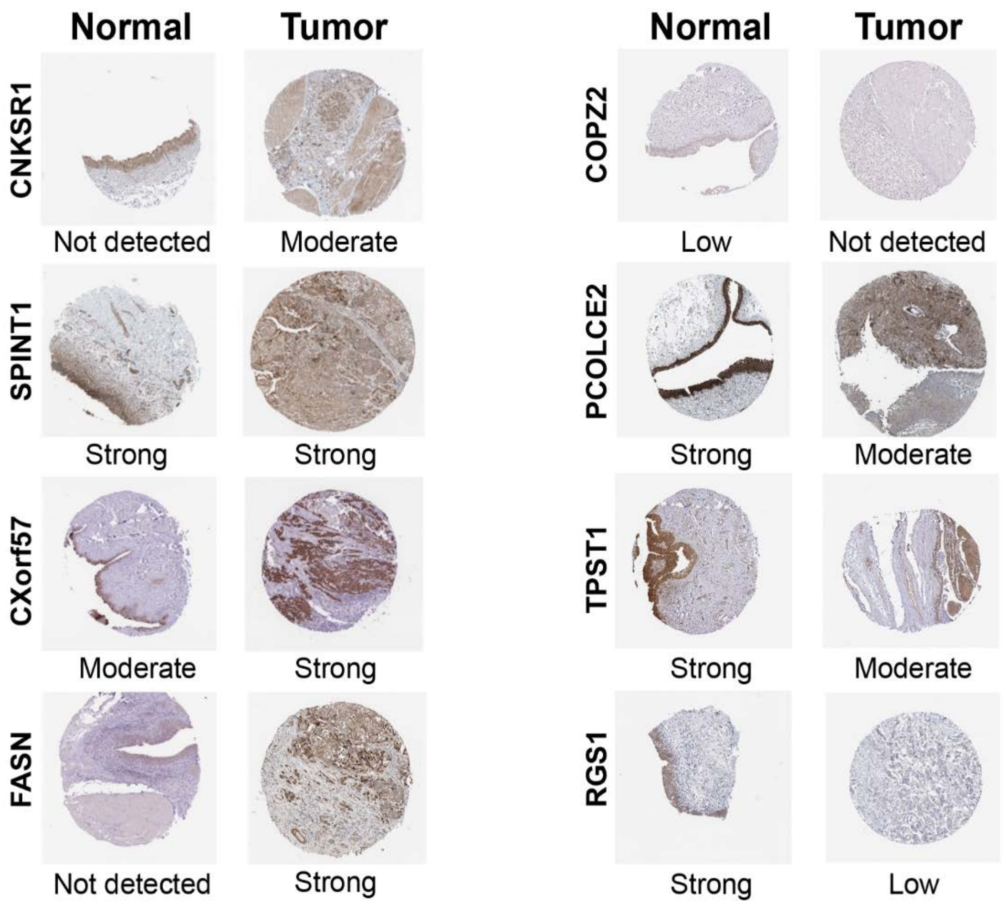

Supplementary Figure 2. Expression levels of the eight genes in tumor tissues and normal tissues. Images were taken from the Human Protein Atlas.
