# Supplementary material for: Identification and validation of a novel signature for prediction the prognosis and immunotherapy benefit in bladder cancer
Source: PeerJ. 2022 Jan 25;10:e12843. doi: 10.7717/peerj.12843 (PMC8796709; doi:10.7717/peerj.12843)
Supplement: Supplemental Information 7 — (A) CNKSR, (B) COPZ2, (C) CXorf57, (D) FASN, (E) PCOLCE2, (F) RGS1, (G) SPINT1, (H) TPST1 [file peerj-10-12843-s007.pdf]

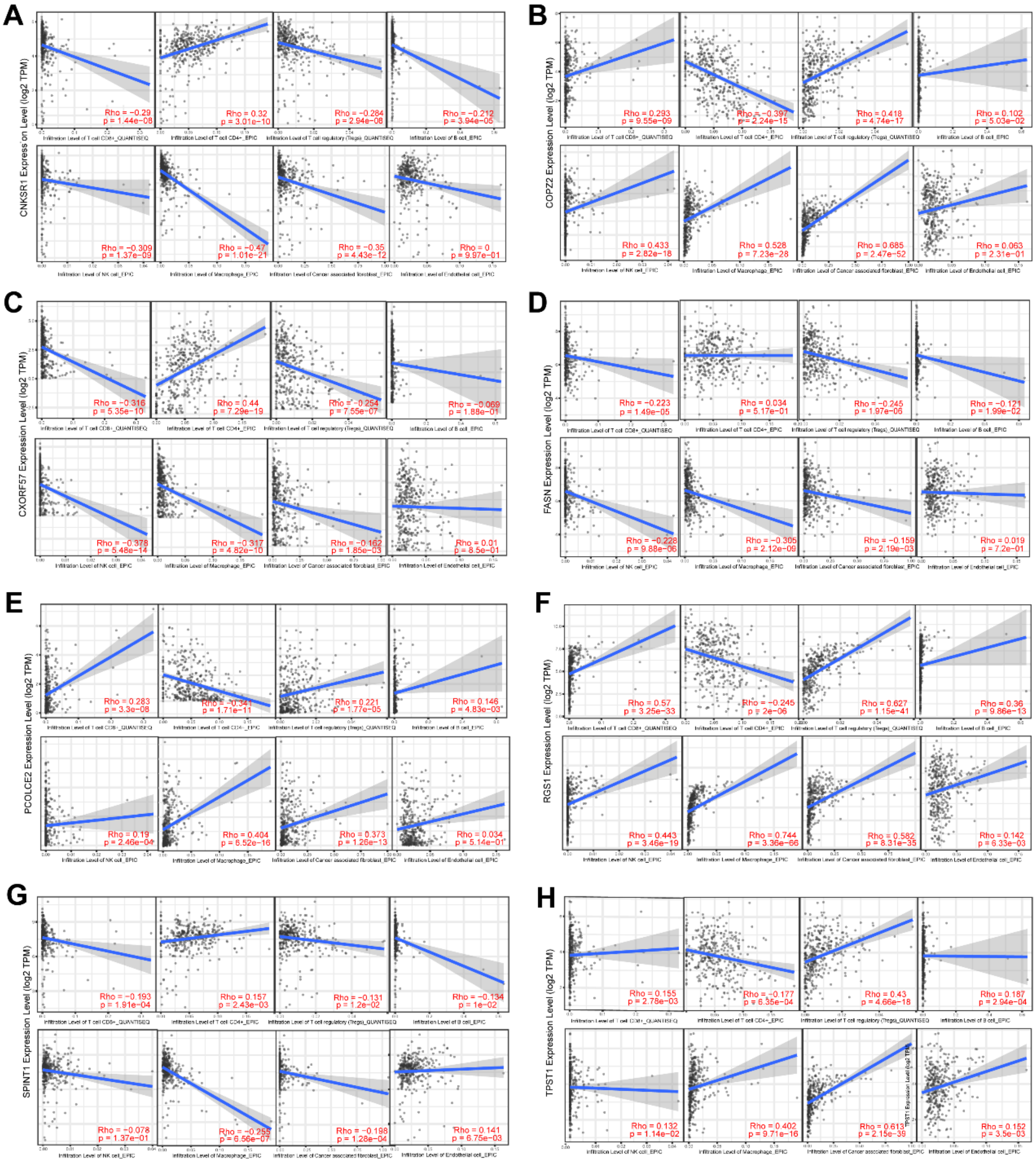

Supplementary Figure 3. Correlation between immune infiltration level and expression of each gene in the gene signature. (**A**) *CNKSR*, (**B**) *COPZ2*, (**C**) *CXorf57*, (**D**) *FASN*, (**E**) *PCOLCE2*, (**F**) *RGS1*, (**G**) *SPINT1*, (**H**) *TPST1*
